# Prevention and Management of Bacterial Infections in Cirrhosis

**DOI:** 10.4061/2011/784540

**Published:** 2011-08-28

**Authors:** Sunil K. Taneja, Radha K. Dhiman

**Affiliations:** Department of Hepatology, Postgraduate Institute of Medical Education & Research, Chandigarh 160012, India

## Abstract

Patients with cirrhosis of liver are at risk of developing serious bacterial infections due to altered immune defenses. Despite the widespread use of broad spectrum antibiotics, bacterial infection is responsible for up to a quarter of the deaths of patients with liver disease. Cirrhotic patients with gastrointestinal bleed have a considerably higher incidence of bacterial infections particularly spontaneous bacterial peritonitis. High index of suspicion is required to identify infections at an early stage in the absence of classical signs and symptoms. Energetic use of antibacterial treatment and supportive care has decreased the morbidity and mortality over the years; however, use of antibiotics has to be judicious, as their indiscriminate use can lead to antibiotic resistance with potentially disastrous consequences. Preventive strategies are still in evolution and involve use of antibiotic prophylaxis in patients with gastrointestinal bleeding and spontaneous bacterial infections and selective decontamination of the gut and oropharynx.

## 1. Introduction

Bacterial infections are a common, recurrent complication of cirrhosis associated with poor outcome [1]. Decompensated cirrhosis has more frequent episodes of infections than compensated cirrhosis. Once infection develops, renal failure, shock, and encephalopathy may follow, which adversely affect survival. Recent prospective studies have shown that 32–34% of cirrhotic patients develop a bacterial infection either at the time of admission or later during their hospitalization [2]. Among cirrhotic patients being admitted for gastrointestinal hemorrhage, the rate of infection is even higher at an estimated 45% and has been shown to be associated with failure to control bleeding and with early variceal rebleeding [3–7]. These numbers contrast sharply with the 5–7% overall infection rates for the general population and emphasize the concept of cirrhosis as an acquired immunodeficient state. The development of infection in cirrhosis is associated with a significantly higher mortality that has been shown to be independent of the severity of liver disease [2, 8–10]. In fact, the in-hospital mortality of cirrhotic patients with infection is approximately 15%, more than twice that of patients without infection. More importantly, infection is directly responsible for 30–50% of deaths in cirrhosis [11].

The mechanisms of increased susceptibility to infections in cirrhosis are unclear. Numerous mechanisms implicated in altered and diminished immunity include increased shunting of blood away from the liver, qualitative dysfunction of the reticuloendothelial system, decreased opsonisation capacity of the ascitic fluid, and increased intestinal permeability of bacteria and associated endotoxins [12]. It has been suggested that there is a role for deficiencies in C3 and C4, downregulation of monocyte human leukocyte antigen-DR expression (and subsequent impaired antigen presentation ability), and impairment of macrophage Fc*γ*-receptor-mediated clearance of antibody-coated bacteria. Patients with alcoholic cirrhosis have depressed neutrophil phagocytic and intracellular killing of microorganisms [13, 14].

The most common infections in cirrhotics are spontaneous bacterial peritonitis (SBP) (25%), followed by urinary tract infection (20%), pneumonia (15%), bacteremia following a therapeutic procedure, cellulitis, and spontaneous bacteremia [1]. Infections are culture positive in 50%–70% of cases. The causative organisms of community-acquired infection are Gram-negative bacilli (GNB), especially *Escherichia coli*, in about 60%, Gram-positive cocci (GPC) in about 30%–35%, and mixed in the last 5%–10%. Nosocomial infections behave differently with 60% GPC and 30%–35% positive for GNB, as a result of the use of therapeutic procedures and previous antibiotic therapies [15]. Beside *Escherichia coli*, the most frequently isolated bacteria are *Staphylococcus aureus*, *Enterococcus faecalis,* and *Streptococcus pneumoniae*. In cirrhotics less virulent organisms cause infections suggesting that, in advanced cirrhosis, bacteria do not need to develop strategies to circumvent host defenses and invade the host [16]. While GNB notably *Escherichia coli* are the causative agents in spontaneous bacterial peritonitis (SBP) and urinary tract infections, Gram-positive bacteria (GPB) predominate in pneumonia (*Streptococcus pneumoniae*) and procedure-associated bacteremia (*Staphylococcus aureus*). Fungal infections especially *Candida* species are involved in up to 15% of severe sepsis in cirrhosis [17].

Most of the available information on bacterial infections in cirrhosis refers to SBP, an entity that is essentially unique to the cirrhotic patient and shares its pathogenesis and management with spontaneous bacteremia and spontaneous bacterial empyema. Gram-positive infections in cirrhosis such as pneumonia or secondary bacteremia are managed according to conventional criteria.

## 2. Spontaneous Bacterial Peritonitis

Spontaneous bacterial peritonitis (SBP), that is, spontaneous infection of ascitic fluid without any apparent intra-abdominal source of infection, is the most characteristic infective complication in cirrhosis [18]. The one-year probability of development of the first SBP in cirrhotic patients with ascites is approximately 10%. This probability is higher in cirrhotic patients with coexisting gastrointestinal bleed, low ascitic fluid protein concentration (<1 g/dl), and/or severe hepatic insufficiency [19–21]. When first described, the mortality of SBP exceeded 90%; however, with early recognition of the disease and prompt and appropriate antibiotic therapy, mortality has been reduced to around 30% [22].

As SBP may pass unrecognized, diagnostic paracentesis should be done in all cirrhotics with ascites on admission to hospital, in-patients with ascites who develop signs of sepsis, hepatic encephalopathy, renal impairment, or altered gastrointestinal motility, and all ascitic patients with a gastrointestinal bleed [23]. SBP is diagnosed with an ascites polymorphonuclear cell count >250/mm^3^, independent of ascites bacteriological culture results [24]. The use of reagent strips may provide a rapid bedside diagnosis of SBP. The test is a quick, safe, and relatively inexpensive screening tool that can be employed at the bedside while awaiting formal cell count and culture analysis. The reagent strip checks for leukocyte esterase activity of activated granulocytes. High numbers of activated leukocytes result in increased hydrolysis of the tested compound and generate a color change on the strip. The results of 8 trials using different types of strips are available [25–32]. Most trials include a very small number of ascites samples with a PMN count >250/mm^3^, and, therefore, although median sensitivity results are ~85%, there is lack of sufficient data for its use in clinical practice unless larger trials validate these observations.

### 2.1. Treatment of Spontaneous Bacterial Peritonitis

Cefotaxime is the most widely studied cephalosporin in patients with SBP and is suitable for empirical therapy for this condition. Prior to 1985, treatment of the condition was suboptimal. A landmark study comparing the combination ampicillin/tobramycin with cefotaxime showed that cefotaxime significantly increased the resolution of bacterial infections, including SBP in cirrhotic patients [33]. Following this study, cefotaxime is considered as one of the first-choice antibiotic therapies in the empirical treatment of SBP in patients with cirrhosis. Recent studies have demonstrated that ceftriaxone is highly effective in the treatment of SBP, with a resolution rate of 100% and a hospital mortality rate of 30% [34, 35]. The combination of amoxicillin and clavulanic acid has also shown to be as effective and safe as cefotaxime in the treatment of SBP [36]. The use of fluoroquinolones for treatment of SBP has shown similar efficacy. Oral ofloxacin has been shown to be as effective as intravenous cefotaxime in the treatment of patients with “uncomplicated” SBP, defined by the absence of gastrointestinal hemorrhage, severe encephalopathy, ileus or septic shock, and a creatinine <3 mg/dL [37].

### 2.2. Spontaneous Bacterial Peritonitis Prophylaxis

The gut appears to be the main source of bacteria that cause SBP and other Gram-negative infections in cirrhosis. Given that SBP is thought to result from the translocation of enteric GNB, the ideal agent should be safe, affordable, and effective at eliminating GNB from the gut while preserving the protective anaerobic flora. Bacterial translocation, the phenomenon by which viable microorganisms from the intestinal lumen migrate to mesenteric lymph nodes and other extraintestinal sites, has been postulated as one of the main mechanisms in the pathogenesis of these infections. Therefore, prophylaxis has been based on the oral administration of nonabsorbable or poorly absorbed antibiotics that will eliminate or reduce the concentration of Gram-negative gut bacteria without affecting Gram-positive organisms or anaerobes, the so-called selective intestinal decontamination. Given the high cost and inevitable risk of developing resistant organisms, the use of prophylactic antibiotics must be strictly restricted to those at highest risk of SBP.

Long-term administration of orally administered norfloxacin, a poorly absorbed quinolone, has been shown to produce a marked reduction of GNB from the fecal flora of cirrhotic patients with no significant effects on GPC or anaerobic bacteria [38]. The development of infections by quinolone-resistant organisms is the main complication of long-term norfloxacin prophylaxis. A recent study showed clear differences in the type of bacteria causing infections in cirrhotic patients on chronic quinolone prophylaxis: while 67% of infections in untreated cirrhotic patients were due to Gram-negative organisms, infections in patients receiving quinolone prophylaxis were mostly due to Gram-positive organisms (79%). This study also showed the emergence of severe nosocomial *Staphylococcal *infections due to methicillin-resistant strains [39]. Therefore, SBP prophylaxis should be considered only in high-risk populations or the patients awaiting liver transplantation.

Three patient populations considered at high risk and in whom prophylactic antibiotic therapy has been recommended are patients with prior history of SBP, patients admitted with gastrointestinal bleed, and patients with low total protein content in ascitic fluid. 

#### 2.2.1. Prophylaxis in Patients with a Previous Episode of Spontaneous Bacterial Peritonitis

The 1-year and 2-year probabilities of survival after an episode of SBP are of 30–50% and 25–30%, respectively, [18]. Therefore, patients recovering from an episode of SBP should be considered as potential candidates for liver transplantation. As such, it is imperative to initiate long-term prophylactic therapy in all patients with prior history of SBP. Norfloxacin, a poorly absorbed quinolone selective to GNB, was shown to decrease the 1-year probability of SBP from 68% to 20% when dosed at 400 mg daily. In this study, the probability of developing SBP specifically from GNB was reduced from 60% to 3% [40]. Subsequently economic analysis studies have shown substantial cost savings in initiating prophylactic therapy in patients with a prior episode of SBP rather than treating at time of diagnosis [41, 42]. Another trial using oral trimethoprim/sulfamethoxazole also showed efficacy in the prevention of SBP. It can be used as an alternative in patients who are unable to take or develop resistance to quinolones [43]. Prophylactic therapy should be instituted after the completion of antibiotics for acute SBP and continued until death, transplant, or resolution of ascites [44].

#### 2.2.2. Prophylaxis in the Setting of Gastrointestinal Bleeding

All cirrhotic patients who develop an upper gastrointestinal bleed are at risk of a variety of bacterial infections, including SBP, within the first few days following the bleed. Bacteria of enteric origin are most commonly implicated, and the development of infection is associated with a poor prognosis [45–47]. Among all hospitalized cirrhotic patients, those admitted specifically with a gastrointestinal hemorrhage have a higher rate of infection than cirrhotic patients hospitalized for other reasons (45% versus 33%). Furthermore those with gastrointestinal hemorrhage complicated by an uncontrolled infection are at substantial risk of rebleeding, difficult to control bleed, and underlying sepsis-associated coagulopathy [48]. A meta-analyses of trials in patients with variceal hemorrhage has shown that antibiotic prophylaxis reduced the incidence of severe infection and decreased mortality [49]. There has been a decrease in mortality from variceal hemorrhage from 43% to 15% over a 20-year period, and antibiotic prophylaxis is independently associated with improved survival [50]. Oral norfoxacin, 400 mg b.d. for at least 7 days, is recommended by the International Ascites Club [44] and oral ciprofoxacin, 500 mg b.d. for 7 days, by the recent British Society of Gastroenterology (BSG) guidelines [51]. The benefit is greatest in those patients with more advanced liver disease. A recent RCT has shown that intravenous ceftriaxone (1 g/day for 7 days) was more effective than oral norfloxacin to prevent severe infections in patients with advanced cirrhosis (characterized by at least two of the following: ascites, severe malnutrition, encephalopathy, or bilirubin >3 mg/dL) and variceal bleeding [52].

#### 2.2.3. Prophylaxis in Patients with Low Ascitic Fluid Total Protein

Ascitic fluid total protein has been shown to be an independent predictor of SBP. The risk of developing SBP in these patients depends largely on ascites protein content. Patients with an ascites protein >1.0 g/dL will not develop SBP in a follow-up period of 2 years, while patients with a low (<1.0 g/dL) ascites protein have a 1-year probability of developing SBP of around 20%. A prospective study in cirrhotic patients during hospitalization found that 15% of patients with ascitic protein <1.0 g/dL developed SBP compared to 2% of those with ascitic protein >1.0 g/dL. The incidence was greatest in those with Child C liver disease and in those who did not receive short-term prophylaxis if admitted with a gastrointestinal bleed. Two non-placebo-controlled studies, which showed a benefit of antibiotic prophylaxis in patients with low ascites protein, included patients with and without prior episodes of SBP and cannot be considered as reliable determinants of primary prophylaxis [43, 53]. Oral norfloxacin administration (400 mg/day) in patients with low protein ascitic levels (<1.5 g/dL) and advanced cirrhosis or impaired renal function without prior SBP episode reduces the probability of SBP and HRS and improved the 3-month survival [54]. Similarly, oral ciprofloxacin (500 mg/day) reduces the 1-year mortality rate in patients with ascitic protein levels <1.5 g/dL and without prior SBP episode [55].

### 2.3. Role of Albumin in Spontaneous Bacterial Peritonitis

In patients with SBP, there is a risk that their systemic hemodynamic parameters can deteriorate, with further arterial and splanchnic vasodilatation. These patients are, therefore, at high risk of developing renal insufficiency [56]. The development of renal failure is the most important indicator of reduced survival in patients with SBP compared with patients without SBP [57]. Renal impairment develops in approximately one-third of patients with SBP and is postulated to arise as a result of a further reduction in effective arterial blood volume, mediated by vasoactive cytokines, with a resultant increased renin-angiotensin-aldosterone system activity [58, 59]. In a multicentre randomized study, 126 patients with SBP were assigned to receive treatment with cefotaxime alone (2 g intravenously every six hours) or cefotaxime plus intravenous albumin. The albumin was given at a dose of 1.5 g/kg in the first 6 h after diagnosis, followed by a further infusion of 1 g/kg on the third day. With the standard treatment, renal impairment developed in 33% of patients, whereas with the combination therapy it occurred in only 10%. The in-hospital mortality rates were 28% and 10%, respectively, [60]. As the development of renal failure in cirrhotic patient with SBP carries a high risk of morbidity and mortality, the use of albumin infusion as an adjunctive therapy in the treatment of patients with SBP will continue until further studies are available.

### 2.4. Role of Probiotics in Prevention of Spontaneous Bacterial Peritonitis

Recent research in the use of certain probiotic agents has shown promise in decreasing cytokine release and improving neutrophil function in cirrhotic patients [61, 62]. The use of probiotics in this setting is attractive not only because of its ability to modulate gut flora in favor of protective anaerobic organisms but also because of its effects in promoting gut barrier function. However, there is no data to support decreasing infection rates or improved outcomes with probiotics in this population. Bacteriotherapy with *Lactobacillus* has been reported to correct bacterial overgrowth, stabilize mucosal barrier function, and decrease bacterial translocation in rat models of acute liver injury and failure. However, the administration of *Lactobacillus acidophilus-* and *Lactobacillus* GG-fermented diets to animals with portal hypertension and cirrhosis failed to show any reduction in bacterial translocation or in ascites infection rates [63, 64].

Two prospective randomized studies demonstrated the efficacy of probiotics in reducing postorthotopic liver transplantation (OLT) infections [65, 66]. In the first study, OLT patients receiving *Lactobacillus plantarum* 299 and fiber had less posttransplant infections than groups receiving selective bowel decontamination. The second study used Synbiotic 2000 in post-OLT patients for 14 days and also found a lower 30-day infection rate. Importantly, no serious adverse effects were noted in either study. As it is a cheap and feasible alternative to selective intestinal decontamination, further studies are needed to evaluate the effect of this combination in other cirrhotic populations.

## 3. Urinary Tract Infections

Urinary tract infections are the most frequent infective complications in cirrhosis. As in the noncirrhotic population, cirrhotics with indwelling catheters are highly predisposed to develop urinary tract infections. The incidence is markedly higher in female than in male cirrhotics [67]. Urinary tract infections in cirrhosis are usually asymptomatic, and bacteriuria alone is found in a high proportion of urinary tract infections episodes in cirrhotics [68]. The majority of infections are caused by Gram-negative bacilli, and, although urine cultures for identification and in vitro sensitivity testing of causative organisms are always recommended, cases requiring immediate therapy should be empirically started on a quinolone or the older but effective cotrimoxazole. These agents are very active against Gram-negative bacteria and reach high concentrations in urine. Other antibiotic regimes might include amoxicillin plus clavulanic acid or an oral cephalosporin [18, 69].

## 4. Pneumonias

Pneumonias are the third most common infections in patients of cirrhosis after SBP and urinary tract infections. Community-acquired infections are the most frequent, although hospitalized patients admitted to intensive care units have high incidence of nosocomial pneumonias due to predisposing factors such as tracheal intubation, esophageal tamponade, or hepatic encephalopathy. Alcoholics are predisposed to chest infections,* Streptococcus* pneumonia being the causative organism in most lower respiratory tract infections [70]. A significant number of cases of pneumonia are caused by other pathogens normally present in the oropharyngeal area, especially anaerobic bacteria or *Haemophilus influenzae*, or by Gram-negative bacilli, particularly *Klebsiella pneumoniae*, mycoplasma and legionella species [70–72]. Antibiotic regimes combining macrolides and one of the following: cefotaxime, ceftriaxone, amoxicillin-clavulanic acid, are the initial treatment of choice although piperacillin-tazobactam or imipenem may also be used in critically ill patients.

Hospital-acquired pneumonia is predominantly caused by Gram-negative bacilli and staphylococci [71, 72]. Although the identification of the responsible organism in hospital-acquired pneumonia is important for selection of antibiotic treatment, the empiric administration of third-generation cephalosporins (i.e., cefotaxime) should be considered as the first choice of antibiotic. Cirrhotic patients with hydrothorax can develop spontaneous bacterial empyema, which is thought to have the same pathogenesis as SBP, since their isolated bacteria are the same [73]. Therefore, patients with spontaneous bacterial empyema may be treated with the same antibiotic regimens.

## 5. Skin and Soft Tissue Infections

Soft tissue infections, particularly lymphangitis of the lower extremities and abdominal wall, are relatively frequent in cirrhotic patients with ankle edema or ascites. *Staphylococcus aureus* and *Streptococcus pyogenes* are the most frequent causative organisms [74]. Empirical antibiotic with Cloxacillin has been considered the first-choice antibiotic, but, considering these causative organisms, amoxicillin-clavulanic acid and ceftazidime may be a more adequate empiric antibiotic treatment. Clindamycin, vancomycin, and teicoplanin are the other antibiotics with broad-spectrum Gram-positive coverage. 

## 6. Meningitis

More commonly reported in alcoholic cirrhosis with high overall is one month case fatality rate exceeding 50%. *Streptococcus pneumoniae*, *Escherichia coli*, and* Listeria *are the commonest pathogens implicated. Signs of meningeal irritation including nuchal rigidity may be a delayed or even absent clinical sign. Mortality is significantly high and may reach up to 80% in Child-Pugh stage C [75, 76]. 

## 7. Bacteremia and Sepsis

Patients with hepatic dysfunction have an increased risk for bacteremia and sepsis [77]. Bacteria may enter the bloodstream by multiple mechanisms and may quickly progress to sepsis and multiorgan failure due to the immune dysfunctions occurring in cirrhotic patients. Although bacteremia may occur secondary to a preexisting infection or recent instrumentation, this group of patients often develops spontaneous bacteremia. Many of these cases may be incited by occult or overt gastrointestinal bleeding, which is known to greatly increase the risk of bacterial infections [78]. A recent Cochrane Database review found that the accumulated data in eight trials demonstrated that antibiotic prophylaxis at time of gastrointestinal hemorrhage had a significant benefit by decreasing mortality and the incidence of bacterial infections [79]. Despite general adoption of bacterial prophylaxis, cirrhotic patients still have a high rate of bacterial diseases, which often progress to sepsis and severe sepsis.

 Given the degree of immune dysfunction and the morbidity of infections, patients with significant cirrhosis who present with, or with probable, bacteremia or sepsis should undergo rapid diagnostic testing and should receive intravenous antibiotics that treat the likely organisms as soon as possible. In septic patients, early antibiotic initiation with the appropriate agents significantly improves outcomes, and this effect is especially important in immune-compromised patients [80, 81]. 

## 8. Catheter-Related Infections

These infections are common in critically ill patients with cirrhosis. These patients may benefit from appropriate hand hygiene, use of chlorhexidine for skin preparation, use of full-barrier precautions during the insertion of central venous catheters, use of the subclavian vein as the preferred site for insertion of the catheter, and the removal of unnecessary central venous catheters [82].

## 9. Conclusion

Patients with chronic liver diseases sustain impairment to their immune systems, which worsens over time and with disease progression. These defects in their host defense lead to augmented risks of bacterial infections and increased morbidity when they are incurred. Providers caring for patients with hepatic dysfunction should have a heightened surveillance for infectious diseases and suspect that one is present with any acute change in a patient's status. With early diagnosis and proper antibiotic treatment, the mortality of bacterial infections has decreased significantly over the years. 
